# Bcl-2 correlates with localization but not outcome in human osteosarcoma

**DOI:** 10.3892/ol.2013.1395

**Published:** 2013-06-13

**Authors:** KLEMENS TRIEB, IRENE SULZBACHER, BERND KUBISTA

**Affiliations:** 1 Department of Orthopaedics, Klinikum Wels-Grieskirchen, Wels A-4600, Austria; 2 Departments of Pathology, University Hospital of Vienna, Vienna A-1090, Austria; 3 Orthopaedics, University Hospital of Vienna, Vienna A-1090, Austria

**Keywords:** osteosarcoma, bcl-2, expression, outcome, localization

## Abstract

bcl-2 is a member of the bcl-2 family that inhibits apoptosis, plays a crucial role in cell viability and is expressed in various types of tumors. With respect to inconsistent results in previous studies, the aim of the present study was to generate a clear hypothesis with regards to the value of bcl-2 expression as a predictive or prognostic factor in human osteosarcoma. The expression of bcl-2 was examined immunohistochemically in 49 patients with high-grade osteosarcoma and the results were correlated with localization, histological response to chemotherapy, survival and the occurrence of metastases. In patients with osteosarcoma, 21/49 cases (43%) were positive for bcl-2 expression and the remaining cases were negative. A significantly higher expression of bcl-2 was observed in central tumors located in the pelvis (83 vs. 37% positive; P<0.05). The bcl-2 expression status revealed no statistically significant correlation with response to chemotherapy, with 57% of patients with bcl-2-positive tumors showing a good response and 43% showing a poor response. No significant difference was observed when comparing survival or occurrence in bcl-2-positive and -negative tumors. In conclusion, the results of the present study indicate that, despite higher bcl-2 expression in central osteosarcoma, the expression in high-grade osteosarcoma is not a reliable prognostic or predictive marker.

## Introduction

Osteosarcoma is the most frequent malignant bone tumor with the majority of cases in the second or third decade of life. In recent years, survival rates have increased from 20 to 70% due to the combination of radical surgery and neoadjuvant chemotherapy. Chemotherapy is stratified post-operatively with regard to response to chemotherapy and tumor size, which are the most accepted prognostic parameters. To date, the pre-operative stratification of neoadjuvant chemotherapy has not been possible due to the lack of prognostic factors at the time of diagnosis. However, a specific number of non-responding patients remain who do not benefit from the combination of these therapies (1,2).

Efforts have therefore been made to identify prognostic factors in osteosarcoma patients that enable stratification of therapy. To date, a number of specific biological factors have been evaluated as potential prognostic factors in osteosarcoma, including the expression of heat-shock proteins, p-glycoprotein and erbB-2 or alterations of p53 (3–7). However, these determinants have yet to be established in daily use for pre- and post-operative stratification of therapy.

bcl-2 belongs to a family of proteins that regulate programmed cell death, i.e. apoptosis by a multi-step intra-cellular mechanism. Specific members of this family inhibit apoptosis, while others, including Bax, induce it (8,9). The proto-oncogene, bcl-2, is an inhibitor of apoptosis and its expression results in increased cell survival by rendering tumor cells resistant to apoptosis. Previous studies have identified bcl-2 expression in human osteosarcoma, lung cancer and metastases, as well as the role of bcl-2 in tumor progression (10–14).

These studies demonstrated the requirement for further studies of bcl-2 expression to evaluate its prognostic and predictive value in human osteosarcoma (10–14).

Therefore, the aim of the present study was to investigate bcl-2 expression in high-grade osteosarcomas to generate a clear hypothesis with regard to the value of bcl-2 expression as a prognostic factor in human osteosarcoma.

## Materials and methods

### Samples

Tissue specimens were obtained from 49 consecutive patients (21 female and 28 male; mean age ± SD, 21.8±12 years; range, 9–53 years) with high-grade (43 grade III and 6 grade II) osteosarcoma at biopsy prior to receiving chemotherapy. The osteosarcomas were located in the femur (25 cases), tibia (16 cases), ileum or sacrum (6 cases) and humerus (2 cases). Following the biopsy, the patients received multi-agent neoadjuvant chemotherapy according to the Cooperative Osteosarcoma Study (COSS) (1) and definitive surgery. The recent (COSS96) chemotherapy protocol consists of one cycle of adriamycin (90 mg/m^2^) immediately following biopsy, two cases of methotrexate (12 g/m^2^) at 3 and 4 weeks following biopsy and a combination of ifosfamid and cisplatin (6,000 mg/m^2^ and 120 mg/m^2^) at 5 and 8 weeks following biopsy. Definitive surgery was performed 10 weeks after the biopsy, with surgical margins that were wide in all cases and defined histologically according to the criteria previously described by Enneking *et al* (15). This study was approved by the ethics committee of Klinikum Wels, Wels, Austria. Written informed consent was obtained from the patients.

### Tumor classification

The resected tumors were analyzed histologically (grades I–VI) for a response to the pre-operative chemotherapy according to the criteria described previously (16), and were classified as follows: grade I, no viable cells; grade II, single tumor cells or one tumor isle <0.5 cm; grade III, <10% viable tumor cells; grade IV, 10–50% viable tumor cells; grade V, >50% viable tumor cells; and grade VI, no effect of chemotherapy. A good response to chemotherapy was considered as grades I–III and a poor response was considered as grades IV–VI (>10% viable cells). Metastases were identified in 12 patients at the time of diagnosis. The mean tumor size was 158±76 ml, the mean serum alkaline-phosphatase level was 237±152 ml and the mean follow-up time was 52±43 months. In total, 21 patients succumbed to their disease and the remaining 28 patients were followed clinically for a mean duration of 83±27 months.

### Immunohistochemistry

The biopsy specimens were fixed in 7.5% formalin and embedded in paraffin. Immunohistochemistry was performed following deparaffination and rehydration of the slides with a monoclonal antibody for 20 h at room temperature; (M0887; isotype IgG1κ; dilution, 1:20; DakoCytomation, Copenhagen, Denmark). The sections were subjected to a wet autoclave pretreatment (10 min, 1 bar) in citrate buffer (0.5 mM, pH 6) for antigen retrieval (17). Biotinylayed secondary reagents and a streptavidin-biotin complex (P0397; DakoCytomation), together with DAB-development (Sigma-Aldrich, St. Louis, MO, USA), were applied for visualization of the immune reaction. Additional negative controls without the primary antibody were performed. Slides from the lymph nodes were used as positive controls, as these cells are known to express bcl-2. The lymph node slides were always positive for bcl-2 expression and were stained together with the osteosarcoma samples. The slides were scored as negative or positive for bcl-2 expression without knowledge of the patient’s data. Consistent with previous studies (6,10) any immunoreaction of the osteosarcoma cells for bcl-2 in a specific specimen was scored as positive.

### Statistical analysis

Data are presented as the mean ± SD unless stated otherwise and were analyzed by SPSS^®^ software (SPSS, Inc., Chicago, IL, USA). The association of bcl-2 expression was assessed by Kaplan Meier analysis (18) and differences were tested for significance by the logrank test. Cox’s regression was used to analyze bcl-2 expression in a multi-centric model including the presence of metastases and response to neoadjuvant chemotherapy. P<0.05 was considered to indicate a statistically significant difference.

## Results

Of the 49 osteosarcomas studied, 21 cases (43%) were classified as bcl-2-positive, and the immunostaining in the majority of these tumors was heterogenous and contained positive areas. There were 28 cases (57%) classified as bcl-2-negative.

In the centrally-located osteosarcomas (spine or ileum), 5/6 (83%) were positive for bcl-2, whereas only 16/43 (37%) of the peripheral osteosarcomas of the long bones were positive (Table I). Therefore, bcl-2 expression was significantly higher in the centrally-located high-grade osteosarcomas (P=0.0343). No correlation was observed between tumor size or alkaline-phosphatase level and bcl-2 expression.

The osteosarcoma patients were divided into bcl-2-positive and -negative groups and compared with regard to the response to pre-operative chemotherapy and the survival outcome. No difference was observed in bcl-2 expression in the primary tumor for patients with metastases at the time of diagnosis. The comparison of survival rates among the 49 patients revealed that the disease-free and overall survival rates for the 21 patients with bcl-2-positive tumors did not differ significantly from those of the 28 patients whose tumors were negative for bcl-2 expression. The overall survival rate was 54% for patients with bcl-2-positive tumors compared with 46% for bcl-2-negative tumors (data not shown). There was no difference in the bcl-2 expression of the osteosarcoma cells in response to chemotherapy, whereby a poor or good response was exhibited by 43 and 57% of bcl-2-positive cases, respectively, and by 55 and 45% of bcl-2-negative cases, respectively.

## Discussion

bcl-2 is an anti-apoptotic protein that protects cells from a variety of apoptotic stimuli, including cytotoxic drugs, irradiation, heat or growth factor withdrawal. The overexpression of bcl-2 has been identified in a variety of human cancers, including breast, colon, ovarian and prostate cancer. Although bcl-2 confers resistance to malignant cells, it does not always correlate with a poor prognosis.

The present study is based on the hypothesis that there is a direct correlation between the response to chemotherapy, patient survival and bcl-2 expression in human osteosarcoma. To date, a number of clinically accepted prognostic factors have been identified for osteosarcoma, including tumor size and site, response to chemotherapy, primary metastases, surgical remission, age and gender (1). However, specific molecular markers have yet to be identified for daily use, despite the investigation of a number of markers for their prognostic or predictive value. The high expression of heat shock protein 72 has been demonstrated to correlate with a good response to neoadjuvant chemotherapy. In addition, p53 expression has been found to significantly correlate with recurrence prognosis, and p-glycoprotein expression has been hypothesized to represent a negative prognostic factor (5,7,9). For other factors, including platelet-derived growth factor-AA, an association with tumor progression has been reported (8). In specific cases, for instance, in the use of HER/erbB-2 or p53, the results remain controversial or not fully concordant (9).

A previous study in human osteosarcoma revealed marked bcl-2 expression in 46% and moderate expression in 35% of all cases (10). In an additional study, bcl-2 expression was compared with the expression of other markers in the primary tumor and lung metastases. A lack of correlation between the primary tumor and metastases was found in 68.5% of the patients without relation to recurrence survival (6). A more recent study hypothesized a role for bcl-xL in osteosarcoma progression (11) and another study in 56 patients reported a negative correlation in the apoptotic index of osteosarcoma cells, which was closely associated with prognosis (12). In addition, bcl-2 expression of 51 (18/35) and 55% (16/29) was reported in two studies, respectively (13,14). The first study demonstrated that an increased bax/bcl-2 protein expression ratio was indicative of osteosarcoma patients with an unfavorable prognosis (13). The second study identified a decreased survival for patients with a nuclear expression for livin, but not for bcl-2 (14).

These studies demonstrate the requirement for further studies of bcl-2 expression to evaluate its prognostic and predictive value in human osteosarcoma (10–14).

In the present study, bcl-2 expression was analyzed in 49 osteosarcoma biopsy samples. Despite the higher expression of bcl-2 in axial osteosarcomas, the results indicate that bcl-2 expression in high-grade osteosarcoma is not a reliable prognostic or predictive marker. Although apoptosis-regulating proteins appear to play a role in tumor progression, it is likely that other apoptosis-regulating proteins are involved in the resistance to chemotherapy and the patient outcome in human osteosarcoma.

## Figures and Tables

**Table I.** Correlation between bcl-2 expression and localization in human high-grade osteosarcoma.

Localization	n	bcl-2 expression, n (%)		P-value
		Positive	Negative	
Central	6	5 (83)	1 (17)	<0.05
Peripheral	43	16 (37)	27 (63)	
